# P-1405. Drivers of Unplanned Pregnancy among Women with HIV in the Northern and Southern United States

**DOI:** 10.1093/ofid/ofae631.1580

**Published:** 2025-01-29

**Authors:** Arianne S Morrison, Florence Momplaisir, William R Short, Aadia Rana, Anandi N Sheth, Rachel Scott, Gweneth Lazenby, Rodney Wright

**Affiliations:** University of Pennsylvania, Philadelphia, Pennsylvania; University of Pennsylvania, Philadelphia, Pennsylvania; University of Pennsylvania, Philadelphia, Pennsylvania; University of Alabama-Birmingham Heersink School of Medicine, Birmingham, AL; Emory University School of Medicine, Atlanta, Georgia; MedStar Health Research Institute, Washington, DC; MUSC, Charleston, South Carolina; Montefiore, Bronx, New York

## Abstract

**Background:**

National estimates from 1986-2015 show that the proportion of unplanned pregnancies among women with HIV (WWH) is high at 78% and that Black/African American women are disproportionately impacted. We aimed to evaluate the prevalence of unplanned pregnancy using a contemporary cohort of pregnant and postpartum WWH (2019-2024), and identify the individual, interpersonal, community and societal factors associated with pregnancy planning.

**Table 1.**
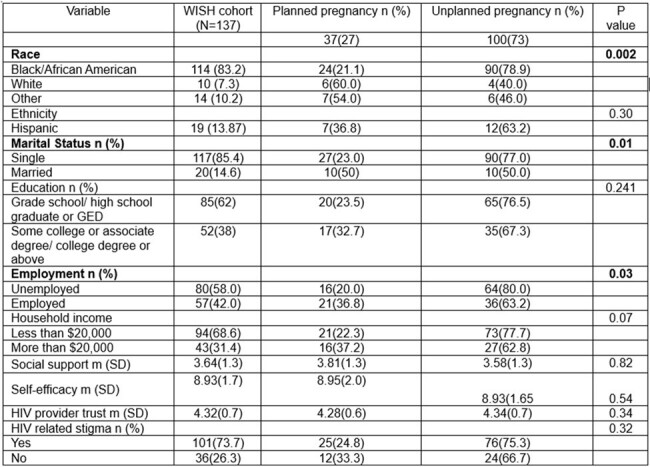
Sociodemographic, sociocultural, and psychosocial variables in a cohort of pregnant and postpartum women (2019-2024) Footnote: social support (Likert scale 1=never 5=always), HIV provider trust (Likert scale 1=strongly disagree 5=strongly agree), self-efficacy (additive scale 0=cannot do at all 10=completely certain can do), HIV related stigma (additive 0=never experienced 9=heavily experienced)

**Methods:**

We performed a secondary data analysis of cross-sectional surveys from an on-going randomized controlled trial testing a peer-led behavioral intervention to improve postpartum retention in HIV care. WWH were recruited from six sites in the northeast and southern US. We used unadjusted and adjusted logistic regression to estimate the association between study outcome, pregnancy planning (“Were you planning to get pregnant?” Yes/No) and individual/interpersonal (self-efficacy, social support, HIV provider trust, HIV related stigma) and community/societal (race, ethnicity, marital status, income, employment) factors.

**Table 2.**
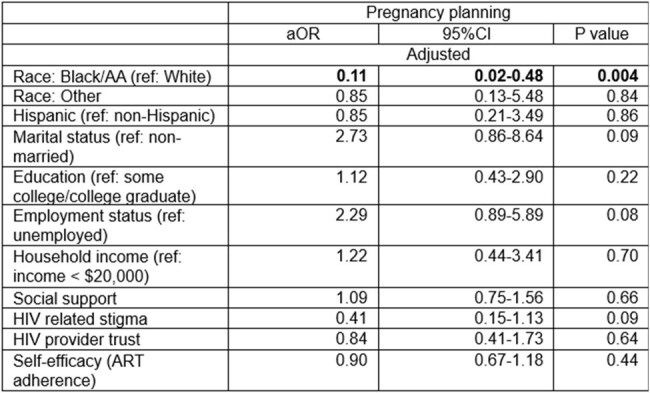
Factors associated with pregnancy planning among women with HIV

**Results:**

To date, a total of 137 women (83% Black, 7% White, 10% Asian, American Indian/Alaska Native, Native Hawaiian/Pacific Islander categorized as other race; 14% Hispanic ethnicity) enrolled in the trial and were included in this analysis. The proportion of unplanned pregnancy was high at 73%. Among WWH with unplanned pregnancy, 90% were Black, 4% were White, 6% were women of other race (p=0.002). While Black women had significantly reduced odds of pregnancy planning (OR 0.18, 95% CI 0.046-0.68), married women (OR=3.33, 95% CI 1.26-8.85), women with higher income (OR=1.27, 95% CI 1.03-1.57) and those with employment (OR 2.33, 95% CI 1.08-5.03) had higher odds of pregnancy planning. In the fully adjusted model, Black race (aOR 0.11 95% CI 0.02 -0.48) was the only factor negatively associated with pregnancy planning.

**Conclusion:**

Unplanned pregnancy among women with HIV and its disproportionate burden on Black women continues to be an area we have yet to make significant progress. Interventions addressing social determinants of unplanned pregnancy and racial disparities are needed to improve reproductive health care for WWH.

**Disclosures:**

**William R. Short, MD**, Gilead: Grant/Research Support|Janssen: Honoraria|ViiV Healthcare: Advisor/Consultant|ViiV Healthcare: Honoraria **Rachel Scott, MD,MPH,FACOG**, DHHS Perinatal Guidelines: Board Member|UW STD Prevention Training Center (UW STD PTC): Honoraria|ViiV Healthcare: Advisor/Consultant|ViiV Healthcare: Grant/Research Support|Vindico CME: Honoraria

